# Chronic Lithium Exposure Reshapes PI3K–mTOR-linked Proteostatic Networks in the Hippocampus of an Alzheimer’s Disease Mouse Model

**DOI:** 10.1007/s12035-026-06030-4

**Published:** 2026-07-06

**Authors:** Caíque de Oliveira Portugal Couto, Maria Luiza Hass das Eiras, João Lucas Juliao de Morais, Carlos Wagner Leal Cordeiro Júnior, Orestes Vicente Forlenza, Vanessa de Jesus Rodrigues de Paula

**Affiliations:** Neuroscience Laboratory LIM 27, Institute of Psychiatry of the Hospital das Clínicas of the Faculty of Medicine of the University of São Paulo, Rua Doutor Ovídio Pires de Campos, 785, Cerqueira César, São Paulo 05403-903 Brazil

**Keywords:** Phosphatidylinositol-3-kinase, Alzheimer's disease, Lithium, Ontological pathways, Neuroplasticity

## Abstract

Alzheimer’s disease (AD) is a progressive neurodegenerative disorder characterized by amyloid-β deposition, tau pathology, and alterations in signaling pathways involved in neuronal survival and protein homeostasis. Lithium has been suggested as a potential neuroprotective treatment, but the molecular mechanisms associated with its long-term effects are still not fully understood. In this study, we investigated the effects of chronic lithium treatment on hippocampal proteins associated with PI3K-related signaling in triple-transgenic Alzheimer’s disease (3xTg-AD) mice. Wild-type and transgenic animals received either a lower or higher lithium dose for eight months. Hippocampal samples were analyzed by LC–MS/MS proteomics followed by protein interaction and functional enrichment analyses. From a total of 7768 identified proteins, bioinformatic analyses identified 157 proteins shared between APP-, MAPT-, and PI3K-associated datasets. Further network analyses identified 18 proteins related to PI3K signaling, including seven proteins shared among all three datasets: FKBP1A, HSPA1B, HSPA8, RAS-related proteins, RPL13, RPL19, and RPL24. These proteins are associated with protein folding, translation regulation, cellular stress responses, and signaling pathways. Chronic lithium treatment was associated with changes in the expression of these proteins in both wild-type and transgenic animals. The observed effects differed between the two lithium concentrations tested and did not follow a simple linear pattern. Our findings suggest that long-term lithium exposure is associated with changes in molecular networks related to proteostasis and translational regulation in the hippocampus. Although additional studies are needed to better understand the mechanisms involved, these results provide a proteomic framework for investigating lithium-sensitive pathways that may be relevant to Alzheimer’s disease.

## Introduction

Alzheimer’s disease (AD) is the most prevalent cause of dementia worldwide and represents a major challenge for aging societies (Xinyi Zhang et al., 2024). The disease is neuropathologically defined by extracellular amyloid-βeta (aβ) plaques and intracellular neurofibrillary tangles composed of hyperphosphorylated tau, which together disrupt synaptic integrity and neuronal survival. Beyond these hallmark lesions, AD is increasingly recognized as a disorder of intracellular signaling and proteostatic imbalance [1].

Among the signaling cascades implicated in AD, the phosphatidylinositol 3-kinase (PI3K) pathway plays a central role in regulating neuronal metabolism, stress responses, protein synthesis, and survival through downstream effectors such as Akt and the mechanistic target of rapamycin (mTOR). Dysregulation of PI3K–mTOR signaling has been linked to both amyloid- and tau-mediated toxicity, positioning this pathway as a potential therapeutic target [2, 3].

Lithium, a long-established mood stabilizer, has been shown to modulate several intracellular pathways relevant to neurodegeneration, including inhibition of glycogen synthase kinase-3β (GSK3β) and regulation of phosphoinositide-dependent signaling pathways involved in neuronal survival, synaptic function, and protein homeostasis [4, 5]. Although experimental evidence suggests neuroprotective effects of lithium in AD models, the molecular networks reshaped by chronic lithium exposure in the hippocampus remain poorly defined. Although lithium has been extensively investigated in Alzheimer's disease models, most previous studies have focused on behavioral outcomes, amyloid and tau pathology, or specific signaling molecules such as GSK3β [6, 7]. In contrast, less is known about the broader proteomic networks that may link lithium exposure to pathways involved in proteostasis, translational regulation, and PI3K-associated signaling. Comparatively less attention has been given to the identification of broader proteomic networks potentially associated with lithium exposure.

In the present study, we applied an integrative proteomic and network-based approach to investigate how long-term lithium treatment modulates hippocampal PI3K-associated protein networks in a triple transgenic mouse model of AD. By focusing on the intersection of amyloid-β and tau signaling with PI3K pathways, we aimed to identify lithium-sensitive molecular processes relevant to proteostasis and neuronal resilience.

## Materials and Methods

### Experimental Animals and Chronic Lithium Administration

All experimental protocols were approved by the local Ethics Committee on Animal Experimentation (CEUA) of the Faculty of Medicine, University of São Paulo (Protocol No. 1877/2023). A cohort of 48 male mice, aged approximately 12 weeks at the onset of the study, was obtained from The Jackson Laboratory. The cohort comprised 24 wild-type B6129SF2/J mice (WT; *n* = 8 per parallel treatment arm) and 24 triple-transgenic Alzheimer’s disease mice [3xTg-AD; B6;129-Psen1tm1Mpm Tg(APPSwe,tauP301L)1Lfa/Mmjax; *n* = 8 per parallel treatment arm]. Male mice were selected exclusively to avoid post-translational and proteomic confounding variables associated with the murine estrous cycle, which is an acknowledged limitation for sex-inclusive translation. Animals were maintained at the Central Animal Facility of the Faculty of Medicine (USP) under strictly controlled environmental parameters (temperature 22 ± 1 °C, relative humidity 50–60%, 12-h light/dark cycle) with ad libitum access to fresh water and specialized solid formulation chows.

The experimental groups were randomized into three dietary treatment arms sustained continuously for 8 months: the Li0 control group, receiving standard solid rodent chow; the Li1 subtherapeutic group, receiving chow supplemented with 1.0 g of lithium carbonate per kilogram (Li2CO3/kg), adjusted based on established preclinical models to target a subtherapeutic serum steady-state threshold; and the Li2 therapeutic group, receiving chow supplemented with 2.0 g Li2CO3/kg to emulate therapeutic serum concentrations.

The lithium concentrations used in the present study were selected based on previous investigations from our group employing the same dietary supplementation protocol in 3xTg-AD mice. In that study, serum lithium concentrations were measured and ranged from 0.16 to 0.21 mmol/L in animals receiving 1 g Li2CO3/kg chow and from 0.26 to 0.35 mmol/L in animals receiving 2 g Li2CO3/kg chow after chronic treatment, confirming distinct systemic lithium exposures between the two treatment regimens. These findings supported the selection of the Li1 and Li2 dietary interventions used in the present work [8].

The selected lithium concentrations were based on previous preclinical studies evaluating long-term lithium administration in rodent models of neurodegeneration. The Li1 diet (1 g Li2CO3/kg chow) was classified as a subtherapeutic dose because it produces serum lithium concentrations below those typically achieved during psychiatric treatment, whereas the Li2 diet (2 g Li2CO3/kg chow) was selected to approximate therapeutic lithium exposure reported in experimental studies. These dose ranges have been previously used to investigate neuroprotective effects of lithium while minimizing toxicity associated with higher systemic exposure [9].

Following the 8-month exposure period, mice were euthanized by rapid decapitation using a calibrated guillotine. This physical method was strictly selected over volatile or injectable anesthetics to minimize rapid post-mortem biochemical artifacts and preserve the immediate stability of target proteostatic systems and protein phosphorylation states. Brain tissue was rapidly extracted from the cranial vault, and the hippocampal structures were microdissected on ice, snap-frozen in liquid nitrogen, and stored at −80 °C until total protein extraction.

### Protein Extraction, Digestion, and LC–MS/MS Proteomics Acquisition

Hippocampal tissue fractions containing 100 ug of total protein were isolated and precipitated via an organic methanol/chloroform protocol. The resulting protein pellets were resuspended in a denaturing buffer of 100 mM Tris–HCl (pH 8.5) containing 8 M urea. Disulfide bonds were chemically reduced with 5 mM tris(2-carboxyethyl) phosphine hydrochloride (TCEP) for 20 min at 37 °C, followed by the alkylation of free cysteines using 25 mM iodoacetamide (IAM) for 20 min at room temperature in the dark. The urea concentration was diluted to 2 M by adding 100 mM Tris–HCl (pH 8.5) to maintain trypsin enzymatic efficiency. Proteins were digested overnight (16 h) at 37 °C using sequencing-grade modified trypsin at a strict 1:100 enzyme-to-protein mass ratio in the presence of 1 mM CaCl2. Proteolysis was quenched with 2% formic acid, and peptide mixtures were kept at −20 °C.

Peptide resolution and mass spectrometry profiling were executed on an HP 1100 Series quaternary HPLC pump online coupled to an LTQ-Velos Orbitrap mass spectrometer equipped with a nano-electrospray ionization source. Chromatographic separations were driven by a 10-h Multidimensional Protein Identification Technology (MudPIT) sequence. In-house packed biphasic fused-silica capillary MudPIT columns (150 um ID/360 um OD) were prepared by sequential loading of 2.5 cm of strong cation exchange (SCX) resin (5 um Partisphere, Whatman) and 2.5 cm of reverse-phase C18 material (5 um ODS-AQ C18, Yamamura Chemical Laboratory). Analytical capillary columns (100 um ID/360 um OD) were packed with 20 cm of C18 material behind a laser-pulled tip.

MudPIT columns were loaded with 20 ug of the digested peptide mixtures and eluted at a stable flow rate of ~300 nL/min using three mobile phases: Solution A (5% acetonitrile, 0.1% formic acid), Solution B (80% acetonitrile, 0.1% formic acid), and Solution C (500 mM ammonium acetate, 5% acetonitrile, 0.1% formic acid). Elution utilized one peptide transfer step followed by seven step-gradients of salt injections (five 60-min steps at 10%, 20%, 30%, 50%, and 70% Solution C; one 120-min step at 100% Solution C; and one final 180-min step combining 90% C/10% B). The mass spectrometer operated in data-dependent acquisition (DDA) mode. Full MS1 precursor scans were collected in the Orbitrap analyzer (300–1200 m/z, resolution 60,000, Automatic Gain Control [AGC] target 5 × 10^5^). The top 20 most abundant precursor ions per scan were isolated and fragmented via Collision-Induced Dissociation (CID) in the linear ion trap (minimum signal threshold of 500 counts, AGC target 1 × 10^4^). Maximum injection times were set to 250 ms for MS1 and 100 ms for MS2. Dynamic exclusion settings included a repeat count of 1, repeat duration of 150 s, exclusion list size of 500, and exclusion duration of 120 s.

### Bioinformatic Filtering Strategy for Candidate Protein Selection

A total of 7768 proteins were identified in the hippocampal proteomic dataset after quality-control filtering and protein-level false discovery rate correction (FDR ≤ 1%). To identify proteins potentially linking Alzheimer’s disease-related pathways with phosphatidylinositol signaling, a stepwise bioinformatic workflow was applied.

First, proteins associated with the PI3K signaling pathway were retrieved from the KEGG Mus musculus reference database. APP and MAPT were subsequently used as Alzheimer’s disease-related anchor proteins for interactome construction in STRING (v12.0), using a minimum confidence score of 0.70.

Independent PI3K–APP and PI3K–MAPT interaction networks were generated and compared using Venny (v2.1). Proteins shared between these networks were retained, resulting in a core set of 157 proteins. This shared protein set was subsequently subjected to STRING network expansion, incorporating the top 50 first-neighbor interactors while maintaining the same confidence threshold.

Functional enrichment analyses identified 18 proteins associated with PI3K-related signaling pathways. Comparison of these proteins with APP- and MAPT-associated datasets revealed seven proteins shared among all three groups. These seven proteins represented the intersection of APP-, MAPT-, and PI3K-associated datasets and were therefore selected as candidate proteins for downstream expression analyses.

Protein abundance was estimated using spectral counting values generated by the IP2 pipeline. To reduce technical variability across samples, abundance values were normalized prior to downstream statistical analyses.

### Statistical Analysis

Protein expression values were quantified using biological replicates (*n* = 8 animals per genotype and treatment group). Statistical comparisons between groups were performed using two-tailed Welch’s *t*-tests, which do not assume equal variance between groups.

For enrichment analyses, p-values were adjusted using the Benjamini–Hochberg false discovery rate (FDR) procedure, and terms with adjusted p ≤ 0.05 were considered significant.

Because expression analyses presented in Figure 2 were restricted to the seven candidate proteins selected through the bioinformatic filtering workflow, significance was evaluated using Welch’s *t*-test and reported as *p < 0.05, **p < 0.01, and ***p < 0.001.

## Results

To investigate proteins that potentially connect Alzheimer’s disease-related pathways to phosphatidylinositol signaling, we analyzed the overlap between proteins associated with the MAPT (tau), APP (beta-amyloid precursor protein), and PI3K pathways identified in the hippocampal proteomic dataset. Using Venny-based intersection analysis, 157 proteins were identified as shared between these pathway-associated datasets. These proteins were subsequently compared between wild-type and 3xTg-AD groups in order to identify proteins potentially associated with PI3K signaling networks related to Alzheimer's disease.

Functional enrichment and protein–protein interaction analyses were subsequently performed to characterize the biological organization of the shared protein set. Gene ontology analysis revealed enrichment of processes related to cellular responses to endogenous stimuli, regulation of catalytic activity, cell migration, and signaling pathways. To further refine the analysis, proteins associated with phosphatidylinositol signaling were identified using the Kyoto Encyclopedia of Genes and Genomes (KEGG) database and integrated with those including tau (MAPT) and beta-amyloid (APP) proteins for STRING network analysis. This approach identified 18 proteins associated with PI3K-related signaling, of which seven were shared between the APP, MAPT, and PI3K-associated datasets.

These shared proteins included FKBP1A, HSPA1B, HSPA8, RAS-related proteins, and ribosomal proteins (RPL13, RPL19, and RPL24), which are functionally associated with protein folding, translation regulation, and stress response pathways.

To identify proteins potentially associated with lithium-related molecular changes within this network, we consulted KEGG. Eighteen proteins related to the PI3K pathway were found, including tau (MAPT) and beta-amyloid (APP) proteins, with a confidence interval of 0.7. These proteins were then used for enrichment in STRING, and analyzed, as described below. Of these, we found 7 shared proteins (FKBP1A → mTOR, HSPs → proteostasis, RPLs → translation), suggesting that these proteins may represent important points of intersection between PI3K-related signaling, proteostasis, and translational regulation.

**Fig. 1:**
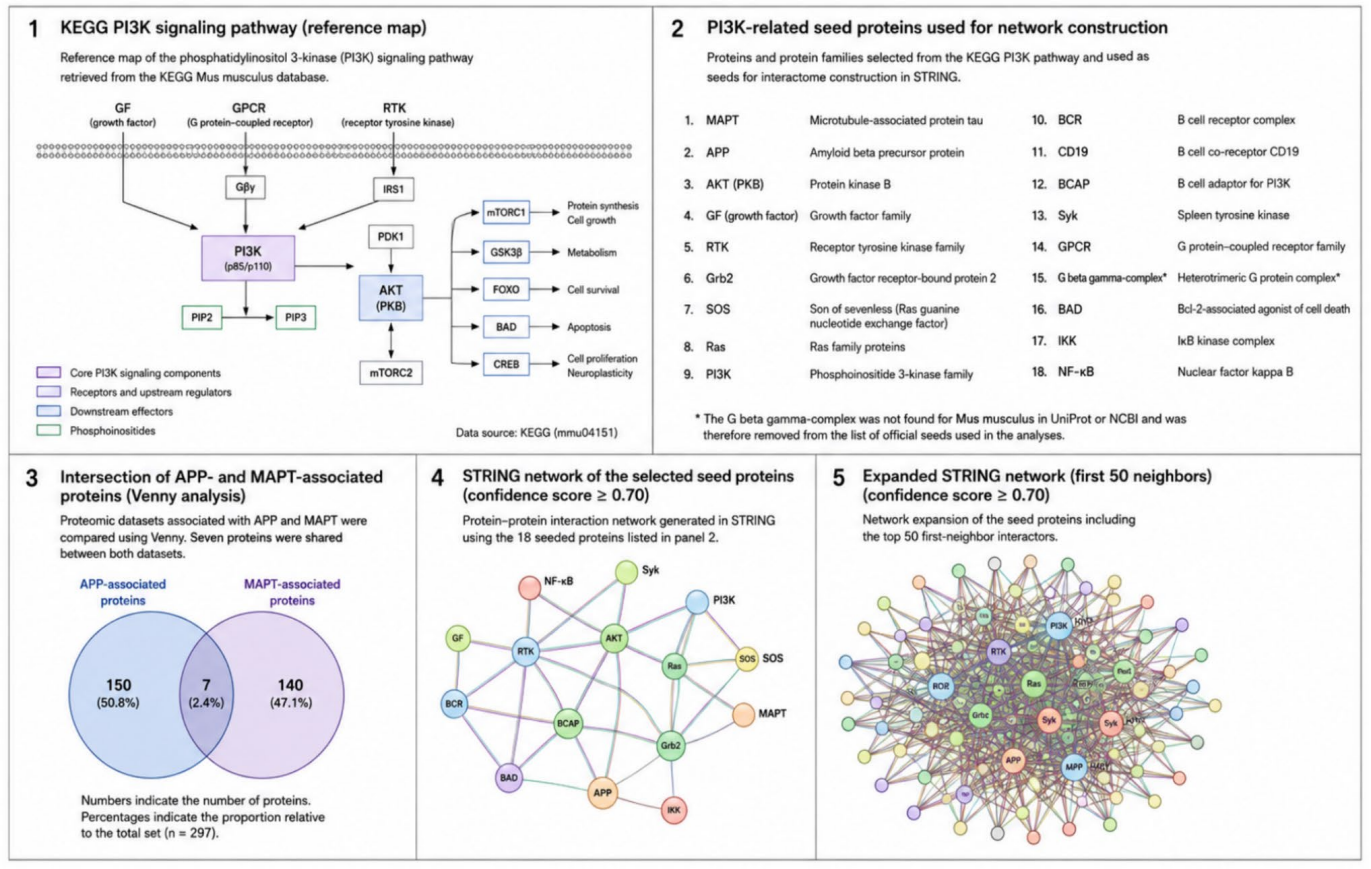
Bioinformatic workflow used to identify proteins linking Alzheimer’s disease-related pathways and PI3K-associated signaling networks. **1** KEGG reference map of the phosphatidylinositol 3-kinase (PI3K) signaling pathway used as the initial pathway source for candidate selection. Highlighted elements represent the PI3K-related components considered during the bioinformatic filtering workflow. **2** PI3K-related seed proteins and protein families selected from the KEGG pathway and used for interactome construction in STRING. GF, growth factor; RTK, receptor tyrosine kinase; GPCR, G protein-coupled receptor. **3** Venny intersection analysis showing proteins shared between APP-associated and MAPT-associated datasets derived from the hippocampal proteomic analysis. Seven proteins were common to both datasets and were retained for subsequent network analyses. **4** STRING protein–protein interaction (PPI) network generated from the selected seed proteins using a confidence score threshold of 0.70. **5** Expanded STRING interaction network including the selected seed proteins and their top 50 first-neighbor interactors, generated using a confidence score threshold of 0.70

In the protein–protein interaction (PPI) analysis, used to identify ontological interactions, only biological pathways with at least four interactions and statistical significance, with a confidence interval of 0.7, were selected. The overall bioinformatic workflow is summarized in Fig. 1. The PI3K reference pathway used for candidate selection is shown in Fig. 11, while the selected seed proteins are presented in Fig. 12. Venny analysis identified seven proteins shared between APP- and MAPT-associated datasets (Fig. 13), which were subsequently analyzed through STRING interaction networks (Fig. 14, 5). We analyzed cellular localization of interactions, molecular functions, and pathways of biological processes as described in Tables 1, 2 and 3.

**Table 1:** Top enriched cellular components identified in the APP/MAPT/PI3K shared protein network

Cellular component					
GO-term	Description	Count in network	Strength	FDR	Biological relevance/interpretation
GO:0035004	Phosphatidylinositol 3-kinase complex, class IA	4 of 4	2.51	8.73e-07	Proteins localize to PI3K class IA complexes, core hubs of PI3K signaling.
GO:0035005	Phosphatidylinositol 3-kinase complex, class IB	4 of 4	2.51	2.37e-07	Association with PI3K class IB complexes supports enrichment of phosphoinositide signaling components.
GO:1990904	Protein-containing complex	41 of 574	0.37	1.70e-07	Indicates that identified proteins participate in multi-protein assemblies and signaling hubs.
GO:0016020	Membrane	56 of 10291	1.20	3.88e-09	Enrichment of membrane localization is consistent with membrane-associated receptor and signaling proteins.
GO:0016021	Integral component of membrane	56 of 9151	1.11	1.64e-09	Supports that many proteins are embedded in membranes, facilitating signal transduction.

**Table 2:** Top enriched biological process identified in the APP/MAPT/PI3K shared protein network

Biological process					
GO-term	Description	Count in network	Strength	FDR	Biological relevance/interpretation
GO:0050896	Response to stimulus	45 of 2040	0.40	2.10e-23	Reflects the involvement of proteins in cellular responses to external and internal cues.
GO:0048583	Regulation of response to stimulus	51 of 4093	0.44	9.53e-24	Indicates regulatory control over adaptive cellular responses.
GO:0050794	Regulation of cellular process	46 of 3148	0.50	1.32e-23	Highlights the role of these proteins in controlling key cellular activities.
GO:0009893	Positive regulation of catalytic activity	45 of 2048	0.73	1.41e-23	Suggests activation or enhancement of enzyme activities, including kinases.
GO:0042325	Regulation of phosphorylation	41 of 1345	1.60	6.00e-20	Strong enrichment for phosphorylation control, consistent with kinase signaling pathways.
GO:1902531	Regulation of intracellular signal transduction	45 of 1401	1.40	1.51e-20	Supports involvement in intracellular signaling cascades including PI3K-related pathways.
GO:0007165	Signal transduction	43 of 5688	0.56	1.28e-20	Confirms that many proteins participate in signal transduction mechanisms.

**Table 3:** Top enriched molecular functions identified in the APP/MAPT/PI3K shared protein network

Molecular function				
GO-term	Description	Strength	FDR	Biological relevance/interpretation
GO:0043548	Phosphatidylinositol 3-kinase binding	1.93	2.84e-15	Direct binding to PI3K indicates functional relevance to PI3K signaling.
GO:0005515	Protein binding	0.39	1.80e-15	Reflects the central role of protein-protein interactions in the network.
GO:0005102	Signaling receptor binding	0.83	5.28e-18	Consistent with interactions with cell surface receptors and signaling molecules.
GO:0030971	Receptor tyrosine kinase binding	1.78	2.79e-19	Supports crosstalk with receptor tyrosine kinase pathways.
GO:0019901	Protein kinase binding	1.06	7.55e-20	Indicates regulation of kinase activity and signaling.
GO:0019900	Kinase binding	1.03	7.55e-20	Broad association with protein kinases.
GO:1990782	Protein tyrosine kinase binding	1.64	7.55e-20	Highlights relevance to tyrosine kinase signaling.
GO:0019899	Enzyme binding	0.76	4.71e-20	General association with enzymes involved in signaling and metabolism.
GO:0051219	Phosphoprotein binding	1.72	3.81e-22	Associates with recognition of phosphorylated proteins.
GO:0001784	Phosphotyrosine residue binding	2.03	2.40e-23	Critical for interactions with phospho-tyrosine motifs.

Gene Ontology enrichment analyses revealed that the shared protein network was predominantly associated with membrane-associated protein complexes, phosphatidylinositol 3-kinase binding, kinase binding, regulation of catalytic activity, and intracellular signaling processes. These findings indicate that the proteins identified through the bioinformatic filtering workflow are functionally organized within signaling and proteostatic networks rather than representing unrelated protein associations. The enrichment of phosphatidylinositol 3-kinase-related molecular functions further supported the selection of these proteins as candidate components linking PI3K-associated signaling with Alzheimer's disease-related pathways.

**Fig. 2:**
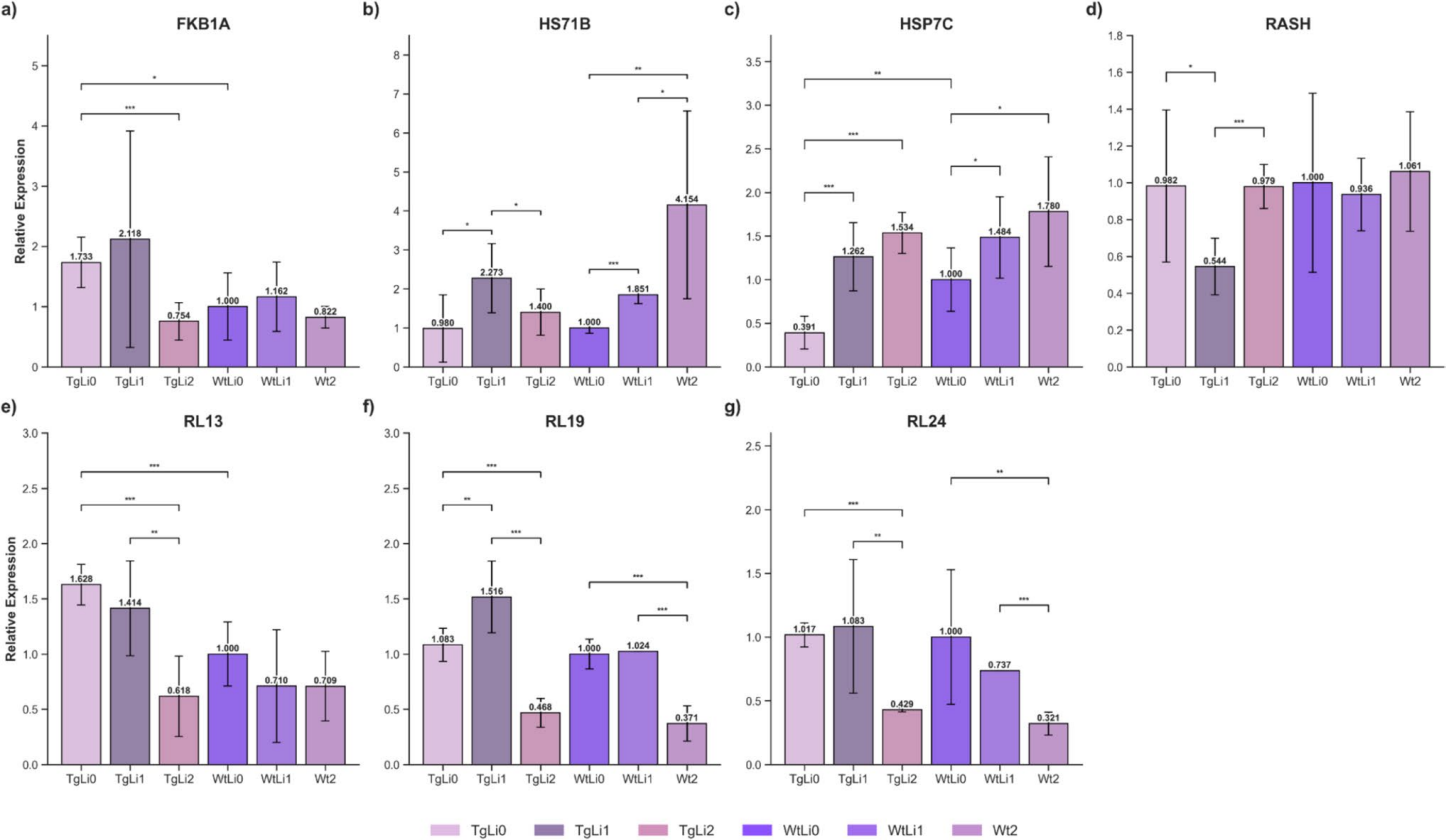
Relative expression of proteins shared between APP, MAPT, and PI3K associated datasets following chronic lithium treatment. **a** FKBP1A; **b** HSPA1B; **c** HSPA8; **d** RAS-related protein; **e** RPL13; **f** RPL19; and (**g**) RPL24. Protein expression values were obtained from the hippocampal proteomic dataset and are presented as relative expression levels across wild-type (WT) and 3xTg-AD (Tg) mice treated with different lithium concentrations (Li0, Li1, and Li2). Statistical comparisons were performed using a two-tailed Welch’s *t*-test. Data are presented as mean ± SD. *p < 0.05, **p < 0.01, ***p < 0.001

Next, we assessed whether chronic lithium exposure was associated with altered expression of the seven proteins shared between the datasets associated with APP, MAPT, and PI3K. Differential expression analysis demonstrated distinct expression patterns at different lithium concentrations in both wild-type and transgenic animals (Figure 2).

These expression profiles differed between the two lithium concentrations tested and did not follow a linear pattern, suggesting selective modulation of proteins associated with proteostatic and translational processes rather than uniform regulation of a single signaling pathway.

## Discussion

The present study identified a small group of proteins shared between APP-, MAPT-, and PI3K-associated datasets that were enriched in functions related to protein folding, kinase regulation, and translational processes. Rather than supporting activation of a single signaling cascade, these findings suggest that lithium exposure may be associated with coordinated modulation of proteostatic networks. The presence of heat shock proteins, ribosomal proteins, and FKBP1A within the shared network is consistent with mechanisms involved in protein quality control and cellular adaptation to stress, processes that are highly relevant to Alzheimer’s disease pathology. The 3xTg-AD mouse model is characterized by the progressive development of both amyloid-β and tau pathology, reproducing two of the major neuropathological hallmarks of Alzheimer’s disease. By approximately 10–12 months of age, these animals typically exhibit substantial intracellular amyloid accumulation, extracellular plaque deposition, hyperphosphorylated tau, and cognitive impairment. Importantly, previous studies have demonstrated that chronic lithium treatment can attenuate amyloid and tau pathology in 3xTg-AD mice, potentially through modulation of signaling pathways involved in neuronal survival, protein homeostasis, and GSK3β activity. These observations support the use of the 3xTg-AD model for investigating molecular mechanisms associated with lithium-responsive pathways in Alzheimer’s disease [6, 7].

Importantly, the present findings should not be interpreted as direct evidence of PI3K/Akt/mTOR pathway activation. The proteins identified in this study were selected through proteomic and network-based analyses and represent molecular associations rather than functional measurements of signaling activity. Therefore, validation of Akt, mTOR, and GSK3β phosphorylation, as well as assessment of amyloid and tau pathology, will be necessary to establish the mechanistic significance of the observed protein alterations.

Seven proteins: FKBP1A, RAS-related proteins, heat shock proteins (HSPA1B and HSPA8), and ribosomal subunits (RPL13, RPL19, and RPL24), emerged as shared nodes connecting PI3K signaling with amyloid and tau pathways. These proteins functionally converge on mTOR activity, rather than demonstrating direct activation of the PI3K/mTOR axis. Current results suggest that lithium exposure is associated with the modulation of interconnected molecular processes related to proteostasis and translational control. This interpretation is corroborated by the identification of heat shock proteins, ribosomal subunits, and signaling components associated with FKBP1A in the shared interaction network [10, 11]. The observation that subtherapeutic and therapeutic doses of lithium produced distinct expression patterns supports the notion that lithium possibly acts through selective network modulation [12].

FKBP1A has been previously implicated in mTOR-associated signaling and protein synthesis pathways. Therefore, its identification in the present dataset may suggest a possible connection between lithium-associated molecular changes and pathways involved in proteostatic regulation. [11, 13]. Heat shock proteins, such as HSPA1B and HSPA8, are involved in protein folding and chaperone-mediated quality control, processes that are critically affected in Alzheimer’s disease due to the progressive accumulation of misfolded proteins [14]. Its identification in the lithium-sensitive network is therefore consistent with the hypothesis that lithium may influence pathways associated with the maintenance of protein homeostasis. The ribosomal proteins identified in this analysis may indicate alterations in the translation machinery and in the regulation of protein synthesis. Growing evidence suggests that dysregulation of ribosomal function contributes to synaptic dysfunction and neuronal vulnerability in neurodegenerative diseases, including Alzheimer’s disease [15].

The present findings should also be considered in the context of the well-established role of glycogen synthase kinase-3β (GSK3β) as a primary molecular target of lithium in Alzheimer’s disease. GSK3β is a downstream effector of PI3K–Akt signaling and a central regulator of both amyloid precursor protein processing and tau hyperphosphorylation. Lithium-mediated inhibition of GSK3β has been consistently reported to reduce amyloid and tau pathology in transgenic AD models, including the 3xTg-AD model [16]. More recently, [4] demonstrated that endogenous lithium deficiency in the brain leads to GSK3β overactivation, driving amyloid deposition, phospho-tau accumulation, microglial pro-inflammatory activation, and synaptic loss in both 3xTg and wild-type mice, effects that were reversed by GSK3β inhibition. These findings are consistent with a central role of the GSK3β–PI3K–Akt axis in mediating lithium's neuroprotective effects. Although GSK3β itself was not among the seven core proteins identified in our proteostatic network, the PI3K-centered remodeling observed here is consistent with upstream modulation of this signaling axis, and future studies incorporating direct assessment of GSK3β phosphorylation would strengthen this interpretation.

Previous studies have strongly implicated lithium-sensitive PI3K/Akt/GSK3β signaling in Alzheimer’s disease models, particularly through the regulation of tau phosphorylation, amyloid precursor protein processing, and neuronal survival [6, 7]. In this context, the present findings do not directly confirm these established mechanisms but provide a complementary proteomic perspective. The identification of FKBP1A, heat shock proteins (HSPA1B and HSPA8), Ras-related proteins, and ribosomal proteins (RPL13, RPL19, and RPL24) within APP, MAPT, and PI3K associated networks suggests that lithium-related molecular changes may also involve broader processes linked to proteostasis, translational regulation, and cellular stress responses. These findings support the view that lithium-responsive mechanisms extend beyond individual signaling molecules such as GSK3β and may involve coordinated regulation of protein quality control and cellular homeostasis, while remaining consistent with the established importance of the PI3K/Akt/GSK3β axis in Alzheimer’s disease.

An important observation was that the proteomic changes associated with lithium exposure did not follow a linear pattern across the tested concentrations. An important observation was that the proteomic changes associated with lithium exposure did not follow a linear pattern across the tested concentrations. Since only two lithium concentrations were evaluated, the present study does not establish a true dose–response relationship. However, distinct effects were observed at the two lithium concentrations tested. This non-linearity may be particularly relevant in the context of long-term treatment strategies aimed at minimizing toxicity while preserving neuroprotective potential.

Several limitations must be considered in interpreting these results. Firstly, the present study is based on proteomic and network association analyses, and does not include direct functional validation of PI3K/mTOR signaling activity, GSK3β regulation, tau protein phosphorylation, or amyloid pathology. Therefore, it is not possible to establish definitive mechanistic conclusions about the activation of these pathways.

Furthermore, since no histopathological analyses were performed, only male mice were analyzed, and behavioral correlates were not evaluated, it remains uncertain whether the observed proteomic alterations are directly related to lithium exposure, are secondary to alterations in Alzheimer’s disease pathology, or are associated with broader cellular responses to stress. The exploratory nature of the present study also limits the causal interpretation of the identified molecular interactions.

Future studies integrating phosphoproteomic approaches, functional validation assays, histopathological analyses, and behavioral outcomes will be important to clarify the biological significance of these lithium-associated proteomic signatures.

Because amyloid-β and tau pathology were not directly quantified, some of the observed proteomic alterations may reflect downstream consequences of altered pathological burden. Therefore, the identified networks should be interpreted as lithium-associated molecular signatures rather than evidence of pathology-independent mechanisms. To our knowledge, this is one of the first studies combining hippocampal proteomics with APP-, MAPT-, and PI3K-centered network analysis to identify lithium-responsive molecular signatures in the 3xTg-AD model.

## Conclusion

**Fig. 3:**
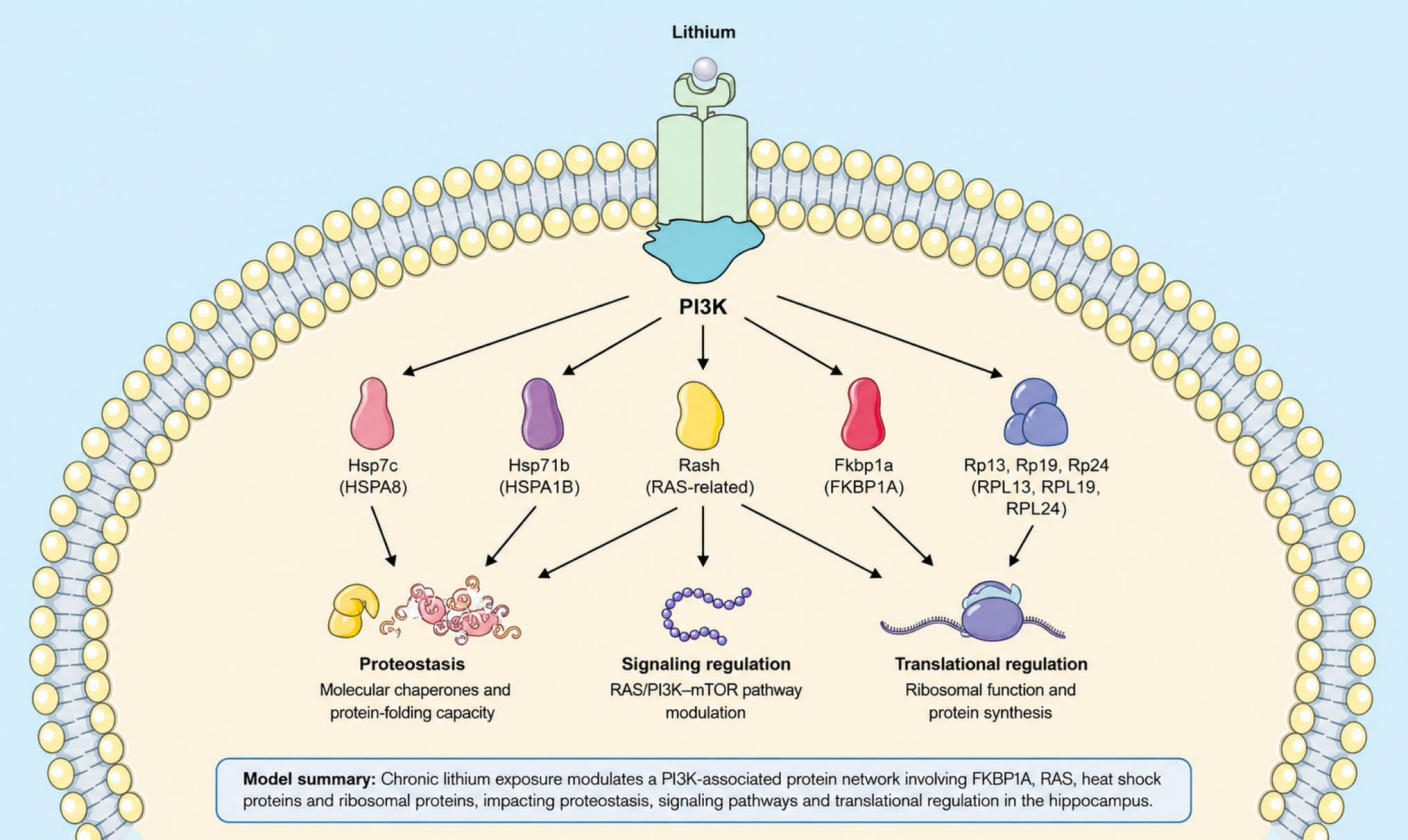
Proposed biological model integrating the principal proteins identified in the APP-, MAPT-, and PI3K-associated network following chronic lithium exposure. The model summarizes the main functional relationships suggested by the proteomic and bioinformatic analyses. Lithium-associated modulation of a PI3K-related protein network was linked to proteins involved in proteostasis (HSPA1B and HSPA8), signaling regulation (RAS-related proteins and FKBP1A), and translational control (RPL13, RPL19, and RPL24). These proteins were identified as shared components of APP-, MAPT-, and PI3K-associated datasets and were enriched in biological processes related to protein folding, kinase-associated signaling, and ribosomal function. The diagram represents a conceptual synthesis of the proteomic findings and does not imply direct causal or experimentally validated mechanistic relationships between the indicated proteins and pathways

In conclusion, chronic lithium exposure was associated with the modulation of proteins linked to PI3K-related proteostatic networks in the hippocampus of 3xTg-AD mice. The identified proteins are functionally related to protein folding, translation regulation, and stress response pathways, suggesting potential intersections between lithium-sensitive molecular processes and central features of Alzheimer’s disease biology. While further validation is needed, these findings provide an exploratory framework for future mechanistic studies investigating lithium-associated signaling networks in neurodegeneration. A conceptual summary of the proposed relationships among the identified proteins is presented in Fig. 3.

## Data Availability

All data supporting the findings of this study are available within the paper.

## Declarations

### Use of Artificial Intelligence

Artificial intelligence (AI)-assisted tools, including ChatGPT (OpenAI), NotebookLM (Google), and Gemini (Google), were used during the preparation of this manuscript to improve English grammar, language clarity, overall readability, and figure presentation. These tools were also used to support text revision and image layout refinement. No AI tools were used to generate, analyze, interpret, or modify the scientific data, results, or conclusions presented in this study. All scientific content, analyses, interpretations, and final editorial decisions were reviewed and approved by the authors, who take full responsibility for the accuracy and integrity of the manuscript.
